# The Effect of Contextual Mobile Advertising on Purchase Intention: The Moderating Role of Extroversion and Neuroticism

**DOI:** 10.3389/fpsyg.2022.849369

**Published:** 2022-05-26

**Authors:** Yajuan Wang, Zhanghua Zhou, Chonghuan Xu, Songsong Zhao

**Affiliations:** ^1^School of Business Administration, Zhejiang Gongshang University, Hangzhou, China; ^2^Zheshang Research Institute, Zhejiang Gongshang University, Hangzhou, China; ^3^Academy of Zhejiang Culture Industry Innovation & Development, Zhejiang Gongshang University, Hangzhou, China

**Keywords:** purchase intention, personality traits, contextual mobile advertising, advertising attitudes, extroversion and neuroticism

## Abstract

Contextual mobile advertising, with the advantages of high interactivity and immersive experience, is the mainstream trend of future Internet advertising. Current studies have explored the benefits of contextual mobile advertising while lacking the analysis of contextual mobile advertising factors on consumer purchase intentions. This study investigates the mechanisms by which the characteristics of contextual mobile advertising evoke consumers' purchase intentions through advertising attitudes to reveal how extroversion and neuroticism in personal traits moderate the relationship between characteristics of contextual mobile advertising and advertising attitudes. Based on a sample of 543 community residents with mobile shopping experience in China, this study uses structural equation modeling to validate the relationships between the variables and draws conclusions. The findings help advertisers to grasp the important characteristics of contextual mobile advertising, improve consumers' attitudes toward advertising, and enhance purchase intentions. Furthermore, it is possible to expand perceptions of the effectiveness of contextual mobile advertising among consumers with different personality traits.

## Introduction

With the rapid development of the Chinese mobile Internet, the size of mobile Internet advertising continues to climb in the percentage of overall Internet advertising. As reported by iResearch in 2020, in China, mobile advertising accounted for 87.7% of all Internet advertising^1^. Such rapid growth is attributed to the increasingly diverse forms of mobile Internet advertising which are not limited by time and space nowadays. The current forms of mobile Internet advertising are mainly derived from the migration of Internet advertising. Contextual advertising is one of the major forms of Internet advertising. It entails the display of relevant advertising based on the content that consumers view, exploiting the potential that consumers' content preferences are indicative of their product preferences (Zhang and Katona, 2012). Compared with other forms of Internet advertising, contextual advertising induced more favorable attitudes toward advertising (Chun et al., 2014). Moreover, through the presentation of contextual advertising, consumers are also generating more purchase intention. Nyström and Mickelsson (2019) introduced the concept of contextual-embedded selling, suggesting that when digital advertising content was thematically aligned with surrounding content, it promoted consumers' purchase intention. Given the advantages of contextual advertising, more and more companies are introducing it into the field of mobile Internet advertising. For example, Amazon, Google (now Alphabet Inc), and Alibaba, massively put contextual mobile ads on mobile apps. Andrews (2017) pointed out that mobile advertising embedded with contextual information could help marketers by delivering the right mobile advertising to the right person at the right time at the right place in the right context. However, in the current contextual mobile advertising delivery, many consumers perceive contextual mobile advertising as interference with their browsing, and thus advertising avoidance occurs. How to improve consumers' attitudes toward contextual mobile advertising and enhance advertising delivery effectiveness as well as how to improve the purchase intention have become popular issues in this research field.

It is worth noting that if companies want to change consumers' advertising attitudes through the presentation of contextual mobile advertising, to arouse more purchase intention and promote the sales of their products or services, they should study what characteristics of contextual mobile advertising have such an effect. At present, scholars do not give a clear definition of contextual mobile advertising. Yuan and Tsao (2003) first mentioned contextual mobile advertising. They pointed out that it was advertising used for advertisers to create tailor-made campaigns targeting users according to where they were, their needs of the moment, and the devices they were using. Some scholars started with location-based mobile advertising research and discussed some characteristics of contextual mobile advertising (Banerjee and Dholakia, 2012; Bauer and Strauss, 2016; Lin and Bautista, 2018; Ryu and Park, 2020). More scholars study contextual mobile advertising from the perspective of contextual information embedded in mobile advertising (Chun et al., 2014; Andrews et al., 2016; Andrews, 2017; Nyström and Mickelsson, 2019). Furthermore, how the important characteristics of contextual mobile advertising affect advertising attitudes and the mechanisms that evoke consumers' purchase intentions are also required in further research. Advertising attitudes refer to the tendency of people to respond positively or negatively to an advertising stimulus in certain specific contexts, reflecting the general evaluation of people's liking or disliking of advertising (Lutz, 1985). Therefore, the important characteristics of contextual mobile advertising can be considered as stimulate under which consumers develop positive or negative psychological response tendencies and form an overall evaluation of the liking or disliking of contextual mobile advertising. Fishbein and Ajzen (1975) and Davis et al. (1989) both argued that attitudes were significant predictors of behaviors or intentions. It can be speculated that consumers' attitudes toward contextual mobile advertising are likely to be antecedents of their purchase intentions.

In addition, a potential factor that cannot be ignored in the process of the important characteristics of contextual mobile advertising influencing advertising attitudes is personality traits. The previous studies of personality traits can help us deeply understand the differences in consumers' attitudes. Different consumers have different advertising preferences, two people will not have the same emotional reactions and attitudes toward advertising; they often respond most positively when advertising matches their personality traits, developing positive advertising attitudes (Myers et al., 2010). Thus, personality traits are integral to explain the effects of individual differences in advertising attitudes. For example, in a study where personality traits moderated advertising effectiveness, Khare and Handa (2009) pointed out that consumer personality traits interacted with different advertising characteristics, and that the consistency between specific personality traits and certain advertising characteristics could encourage positive consumer attitudes, brand memory, and purchase intentions of advertising products. Furthermore, it has been suggested that personality should not be understood as an independent variable, but rather as a mediating or moderating factor in consumer surveys (Myers et al., 2010; Uribe et al., 2021). Since consumer personality traits interact with different advertising characteristics (Khare and Handa, 2009), this study suggests that some specific traits in personality traits may moderate the relationship between certain characteristics of contextual mobile advertising and advertising attitudes.

Above all, this study attempts to investigate the processes and mechanisms by which relevant characteristics of contextual mobile advertising evoke consumers' purchase intentions through advertising attitudes in a sample of Chinese community residents with the online shopping experience, and discusses how personal traits moderate the relationship between significant characteristics of contextual mobile advertising and advertising attitudes. First, this study conducted a literature review, selected content accuracy and contextual interaction as important characteristics of contextual mobile advertising, discussed the relationship between relevant variables theoretically, and proposed hypotheses to construct a theoretical model. Second, questionnaire surveys and measurement of the relevant variables were conducted. Finally, structural equation modeling was used to verify the relationship among the variables and to draw conclusions. The results of our study help advertisers grasp the important characteristics of contextual mobile advertising, improve consumers' attitudes toward advertising, and enhance purchase intentions. At the same time, it is also possible to expand consumers' perceptions of the effectiveness of contextual mobile advertising among consumers with different personality traits.

## Literature Review and Hypotheses

### Content Interaction of Contextual Mobile Advertising and Advertising Attitudes

In the study of mobile advertising acceptance, Liu et al. (2019) concluded that interactivity was a key factor influencing attitudes. Interactivity can increase the potential enjoyment experience and help users form more positive attitudes toward advertising (Ayesha, 2013), for example, Plume and Slade (2018) studied consumers' motivation to share sponsored advertising in a travel context, where interactivity was a significant driver for sharing travel-related sponsored advertising on Facebook. In low-interactivity contexts, consumers show higher avoidance of online advertising (Jin and Villegas, 2007). Interactive advertising is more effective than non-interactive advertising in attracting users' attention (Yang et al., 2016). Meanwhile, advertising supported by social context is effective in grabbing users' attention, increasing the click-through rate, boosting memory of the product, and positively influencing users' advertising engagement and acceptance intentions (Ayesha, 2013). In addition, it has been indicated that communication among peers also makes them less critical of Facebook advertising, enhances their engagement with advertising, and weakens their advertising avoidance behavior (Seounmi and Kim, 2019). The studies above largely suggest that content accuracy may be beneficial to contextual mobile advertising to attract consumers' attention and change their attitudes. Thus, the following hypothesis is proposed:

*H1: As contextual interaction increases, advertising attitudes will become more positive*.

### Content Accuracy of Contextual Mobile Advertising and Advertising Attitudes

Content accuracy refers to the precise matching of the graphics, content, text, and editorial formatting of contextual mobile advertising to the information viewed by consumers. Advertising with content accuracy characteristics is more informative and less intrusive than traditional advertising, resulting in more positive attitudes toward advertising (Sweetser et al., 2016). For example, Moore et al. (2005) showed that content accuracy helped consumers to absorb advertising into their activated media schemas more easily, and thus a more positive attitude of consumers toward advertising with high content accuracy. Salem et al. (2018) studied the effect of mobile advertising on the purchase intention of Saudi Arabia consumers and found mobile advertising acceptance might be influenced not only by the mobile media acceptance of consumers but the relevance of advertising content. The studies above largely suggest that contextual interaction may be beneficial to contextual mobile advertising to integrate with the media environment, reducing disruption to users, and inducing more positive advertising attitudes. Thus, the following hypothesis is proposed:

*H2: As content accuracy improves, advertising attitudes will become more positive*.

### Advertising Attitudes and Purchase Intention

Attitudes were positive or negative evaluative propensity to a given person or object (Lien and Cao, 2014). When studying consumer attitudes, Adams and Green (1965) pointed out that consumer attitudes came into the domain of consumer subjective consciousness, which were influenced by internal characteristics and any other objectively political, economic, and social factors. Moreover, consumer attitudes in turn influence their behaviors and purchase intentions. Advertising attitudes can be perceived as subjective consciousness when people watch advertising in a consumption context, consisting of three aspects: cognition, emotion, and behavioral intentions. Cognition refers to the beliefs in advertising, including the learning, thinking, and conception of advertising content; emotion mainly refers to the degree of preference for advertising; behavioral intention refers to the action to be taken after watching the advertisement (Sears et al., 1991). The Theory of Planned Behavior (TPB) proposed by Fishbein and Ajzen (1975) argues that attitudes are important predictors of behavior or intentions, and reflect consumers' favorable or unfavorable evaluations of expected behaviors or intentions. The Technology Acceptance Model (TAM) proposed by Davis et al. (1989) also suggests that consumer attitudes influence consumer behavioral intentions and further influence consumer behaviors. Purchase intention is the likelihood that consumers are planning or ready to purchase products or services in the future (Wu et al., 2011). Grewal et al. (1998) pointed out that purchase behavior and purchase intention were measurements of advertising effectiveness. These studies above largely suggest that consumers' attitudes toward contextual mobile advertising are likely to influence their purchase intentions. Thus, the following hypothesis is proposed:

*H3: As advertising attitudes become more positive, purchase intentions will increase*.

### Moderating Role of Extroversion and Neuroticism

Extroversion indicates the extent to which someone shifts their basic orientation to the outside world, and extroverts are generally sociable (Uribe et al., 2021). Extroverts are strongly associated with positive emotions (Rothbart, 2011) and experience greater joy and well-being compared with introverts. Besides, extroverts are also more likely to engage in challenges instead of escaping or avoiding them (Carver and Connor, 2010). People with higher extroverted traits are more likely to rate non-traditional forms of advertising (Lee and Cho, 2010). In addition, it is demonstrated that extrovert individuals are happier by way of social interactions (Hampson, 2012). Extroverts tend to be more intrigued by new virtual technologies (smart glasses), and they are mainly motivated by the opportunity to assimilate with others (Rauschnabel et al., 2015). For example, Uribe et al. (2021) found that there still existed experience difference in AR advertising owing to different consumer personality traits. Extroversion positively moderated the relationship between advertising contact and advertising acceptance, manifesting more favorable entertainment and informational advertising perception. It can be speculated that contextual mobile advertising embodied with interactive characteristics can create a sense of intimacy among consumers, enhance the perceived pleasure, and thus improve the advertising attitudes.

By comparison, neuroticism refers to emotional stability and the degree to which someone is nervous and insecure (Uribe et al., 2021). Individuals with high neuroticism tend to have faster and stronger emotional responses and are more likely to be moody compared with those with emotional stability (Puechlong et al., 2020). Winter et al. (2021) found that advertising effectiveness was more significant when consumer personality traits matched the content of the advertising. For example, some studies show that social media advertising is more likely to make users feel a sense of relevance to the advertising to them and thus respond positively due to the less detectable and accurate marketing mode based on user data (Voorveld and Van, 2014). Aribarg and Schwartz (2020) also verified through experiments that native advertising with high visual similarity to editorial content could get more clicks compared with regular advertising. Contextual mobile advertising renders relevant advertising according to consumer interests, which emphasizes the consistency of advertising content with the media environment. It can be speculated that highly neurotic individuals are more sensitive and demanding of the accuracy of contextual mobile advertising content compared with other personal traits. When contextual mobile advertising content can accurately match consumer interests, neurotic individuals may change a lot in an emotional state, which may influence advertising attitudes.

Therefore, the relationship between contextual interaction and advertising attitudes will be stronger for individuals with extroversion traits than for neuroticism traits; and the relationship between content accuracy and advertising attitudes will be stronger for individuals with neuroticism traits than for extroversion traits. The following hypotheses are proposed in this study.

*H4a: Extroversion will moderate the relationship between contextual interaction and advertising attitudes. Thus, those high in extroversion will show more positive advertising attitudes toward contextual mobile advertisements with high contextual interaction*.
*H4b: Extroversion will moderate the relationship between content accuracy and advertising attitudes. Thus, those high in extroversion will show more positive advertising attitudes toward contextual mobile advertisements with high content accuracy*.
*H5a: Neuroticism will moderate the relationship between contextual interaction and advertising attitudes. Thus, those high in neuroticism will show more positive advertising attitudes toward contextual mobile advertisements with high contextual interaction*.
*H5b: Neuroticism will moderate the relationship between content accuracy and advertising attitudes. Thus, those high in neuroticism will show more positive advertising attitudes toward contextual mobile advertisements with high content accuracy*.

**Figure 1:**
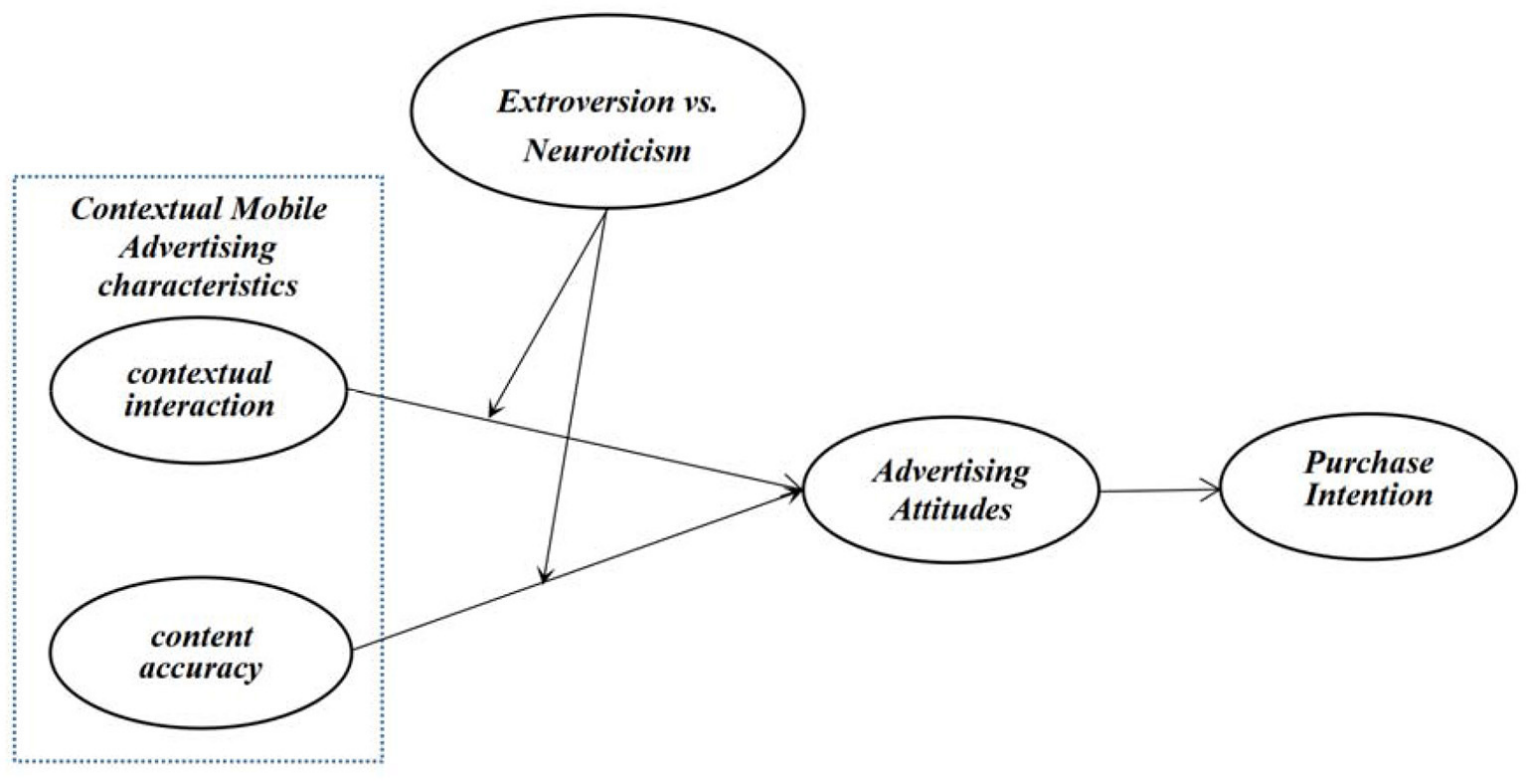
The theoretical model.

Based on the literature review and hypotheses above, the theoretical model of this study is proposed in Figure 1.

## Materials and Methodology

### Participants and Procedure

Data were collected from 24 March to 20 April 2022. The sample was recruited from Chinese community residents who had experience in online shopping and were paid $5 per person. The questionnaire was distributed online through a survey website^2^ The first page of the questionnaire contained the study consent. The next page contained demographic questions, a personality traits test, and a set of self-report questionnaires. To initiate participants' perceptions of contextual mobile advertising, images and descriptions of non-infringing contextual mobile advertising were also presented in the questionnaire.

Participants (*n* = 1,473) were recruited from Chinese community residents with a mobile shopping experience. In this study, the answer to the questionnaire with missing data was not submitted successfully, so there were no missing data. In addition, we screened out the data of extroverts and neurotics from 1,473 respondents through the personality traits test, which was used in the final analysis of this study. The final valid sample (*n* = 543) consisted of 262 females (48.25%) and 281 males (51.75%). The sample had a mean age of 24.72 years (range 18–45). Education was assessed through four options, and participants were asked to check a box to make a choice: 11.42% selected “High school or below,” 9.39% selected “Associate degree,” 46.78% selected “Bachelor degree,” 32.41% selected “Graduate degree or above,” and no one left the question blank. In addition, among the final valid sample (*n* = 543), 295 samples were extroverts and 248 samples were neurotics after the personality traits test. Among them, 152 samples were high extroverts based on 1sd above the mean, and 116 samples were high neurotics based on 1sd above the mean (see Table 1).

**Table 1:** Sample profile.

**Characteristics**	**All**		**Extroverts**				**Neurotics**			
			**High**		**Low**		**High**		**Low**	
	***N***	**%**	***N***	**%**	***N***	**%**	***N***	**%**	***N***	**%**
**Gender**										
Male	281	51.75	92	60.53	59	41.26	37	31.90	45	34.09
Female	262	48.25	60	39.47	84	58.74	79	68.10	87	65.91
**Age**										
18–29	363	66.85	75	49.34	25	17.48	35	30.17	56	42.42
30–39	132	24.31	54	35.53	63	44.06	63	54.31	32	24.24
40–45	48	8.84	23	15.13	55	38.46	18	15.52	44	33.34
**Education**										
High school or below	62	11.42	22	14.47	54	37.76	9	7.76	12	9.09
Associate degree	51	9.39	30	19.74	43	30.07	18	15.52	45	34.10
Bachelor degree	254	46.78	67	44.08	31	21.68	32	27.59	52	39.39
Graduate degree or above	176	32.41	33	21.71	15	10.49	57	49.13	23	17.42
**Total**	543	100	152	100	143	100	116	100	132	100

### Measures

#### Contextual Interaction Scale (CIS)

The Contextual Interaction Scale is a three-item, self-report inventory that was created for this study by adapting a questionnaire developed to measure the perceived interactivity of mobile advertising (Gao et al., 2010). The items utilized were a five-point Likert Scale from not at all (1) to very much (5) to assess participants' perceptions of contextual interaction of contextual mobile advertising, for example, “I like interacting with others in contextual mobile advertising,” “I like receiving social support in contextual mobile advertising,” and “I want to receive responses from others in contextual mobile advertising.” Higher scores reflected a higher perceived level of contextual interaction with contextual mobile advertising. The scale had acceptable internal consistency in this sample (α = 0.84).

#### Content Accuracy Scale (CAS)

The Content Accuracy Scale is a three-item, self-report inventory that was created for this study by adapting a questionnaire developed to measure content-related factors and location-based mobile advertising (Lin and Bautista, 2018). The items utilized were a five-point Likert Scale from not at all (1) to very much (5) to assess participants' perceptions of the content accuracy of contextual mobile advertising. For example: “contextual mobile advertising shows products or services that are like the information I am viewing,” “contextual mobile advertising shows products that satisfy my interests,” “contextual mobile advertising shows products that fit my lifestyle.” The higher scores reflected a higher level of perceived contextual interaction with contextual mobile advertising. The scale had acceptable internal consistency in this sample (α = 0.91).

#### Advertising Attitudes Scale (AAS)

The Advertising Attitudes Scale is a three-item, self-report inventory that was created for this study by adapting a questionnaire developed to measure Advertising Attitudes (Fishbein and Ajzen, 1975). The items utilized were a five-point Likert Scale from not at all (1) to very much (5) to assess participants' perceptions of attitudes to contextual mobile advertising. For example, “contextual mobile advertising is attractive to me,” “I would ignore contextual mobile advertising,” “I like contextual mobile advertising.” The higher scores reflected a higher level of perceived contextual interaction with contextual mobile advertising. The scale had acceptable internal consistency in this sample (α = 0.88).

#### Personality Traits Scale (PTS)

The Personality Traits Scale is a self-report scale with 24 items inventory and is mainly adapted from the IPIP scale which was developed by Goldberg (1999). In the five sub-scales, the extroversion sub-scale (EXS) contains five items, and the neuroticism sub-scale (NES) contains four items. The items utilized were a five-point Likert Scale from not at all (1) to very much (5) to assess participants' personality traits. For example: “I am an enthusiastic person,” “I love group activities,” “I love socializing and interacting,” “I am an active person,” “I always maintain positive emotions.” The higher scores reflected high personality traits. The scale had acceptable internal consistency in this sample (α = 0.83). The extroversion sub-scale had acceptable internal consistency in this sample (α = 0.86), and the neuroticism sub-scale had acceptable internal consistency in this sample (α = 0.85).

#### Purchase Intention Scale (PIS)

The Purchase Intention Scale is a three-item, self-report inventory that was created for this study by adapting a questionnaire developed to measure Millennials' (Millennials cohort) purchase intention of products shown in Facebook advertising (Dehghani and Tümer, 2015). The items utilized were a five-point Likert Scale from not at all (1) to very much (5) to assess participants' perception of attitudes to contextual mobile advertising. For example, “After seeing contextual mobile advertising, I may buy the products or services recommended by the advertising,” “After seeing contextual mobile advertising, I will take the products recommended into my purchase options,” and “After seeing contextual mobile advertising, I want to know more about the products.” The higher scores reflected high purchase intention. The scale had acceptable internal consistency in this sample (α = 0.87).

## Analyses and Results

### Descriptive Statistics

IBM SPSS Statistics 22.0 was used to describe samples' descriptive statistics and the Pearson's correlation coefficient. Three demographic variables were included in this study which were gender, age, and education. After cleaning the data, the total numbers of respondents included in the study analysis were 543. The analyses revealed that there was no significant impact of these demographic variables on the main model variables. Table 2 depicts the mean, standard deviation, and correlations among the study variables. Contextual Interaction (M = 3.56, SD = 0.42), Content Accuracy (M = 3.42, SD = 0.38), Advertising Attitudes (M = 4.12, SD = 0.53), Purchase Intention (M = 3.78, SD = 0.49), Extroversion (M = 2.87, SD = 0.39), and Neuroticism (M = 3.95, SD = 0.67).

**Table 2:** Descriptive statistics and correlations among variables.

**Variables**	**Mean**	**SD**	**CI**	**CA**	**AA**	**PI**	**EX**
CI	3.56	0.42					
CA	3.42	0.38	0.45^**^				
AA	4.12	0.53	0.37^***^	0.27^**^			
PI	3.78	0.49	0.42^***^	0.34^**^	0.22^***^		
EX	2.87	0.39	0.52^***^	−0.25^**^	0.17^**^	0.33^**^	
NE	3.95	0.67	−0.23^**^	0.38^***^	−0.32^**^	−0.27^**^	−0.43

*N = 543, CI, Contextual Interaction; CA, Content Accuracy; AA, Advertising Attitudes; PI, Purchase Intention. EX, Extroversion; NE, Neuroticism*.

** *p < 0.01*,

*** *p < 0.001*.

The existence of common method bias in the data set was tested by using the Harman's one-factor test. The items of all four factors (e.g., contextual interaction, content accuracy, advertising attitudes, and purchase intention) were combined into a single factor and compared with that of the four-factor model. The goodness of fit indices of the one-factor model (X^2^ = 1832.43, df = 578, *p* < 0.01, RMSEA = 0.15, CFI = 0.64, TLI = 0.62, SRMR = 0.07) were significantly poorer than those of the four-factor model (X^2^ = 878.56, df = 556, *p* < 0.01, RMSEA = 0.04, CFI = 0.86, TLI = 0.92, SRMR = 0.05) suggesting that common method bias is not a serious concern in our data set.

### Confirmatory Factor Analysis

Data on contextual mobile advertising characteristics (contextual interaction and content accuracy), advertising attitudes, purchase intention, and personality traits (extroversion and neuroticism) were collected at one time, therefore, it was necessary to conduct Confirmatory Factor Analysis (CFA) to compare different models. The Cronbach's alpha and CFA of the scales were calculated by Mplus 7.0.

The fit of the six-factor model was then compared with two alternative models. The four-factor model (contextual mobile advertising characteristics, advertising attitudes, purchase intention, and personality traits) was estimated in this study. Another model (three-factor) was also estimated, in which contextual mobile advertising characteristics were combined on one-factor, advertising attitudes and purchase intention were combined on one-factor, extroversion and neuroticism were combined on another factor. The fit indices of these alternative models were weak compared to the six-factor model, providing support for the distinctiveness of the model used in this study. The CFA results revealed that the six-factor structure provided a better fit (X^2^ = 436, df = 203, CFI = 0.93, GFI = 0.92, IFI = 0.94, RMSEA = 0.05, SRMR = 0.04) as compared to the alternative models (see Table 3).

**Table 3:** Confirmatory factor analysis results of each variable's construction distinctiveness.

**Model**	**X^**2**^**	**df**	**X^**2**^/df**	**CFI**	**GFI**	**IFI**	**RMSEA**	**SRMR**
Six-factor model	436	203	2.18	0.93	0.92	0.94	0.05	0.04
Four-factor model	544	216	2.35	0.84	0.88	0.86	0.07	0.06
Three-factor model	557	218	2.44	0.82	0.84	0.83	0.09	0.08

### Path Analysis

**Figure 2:**
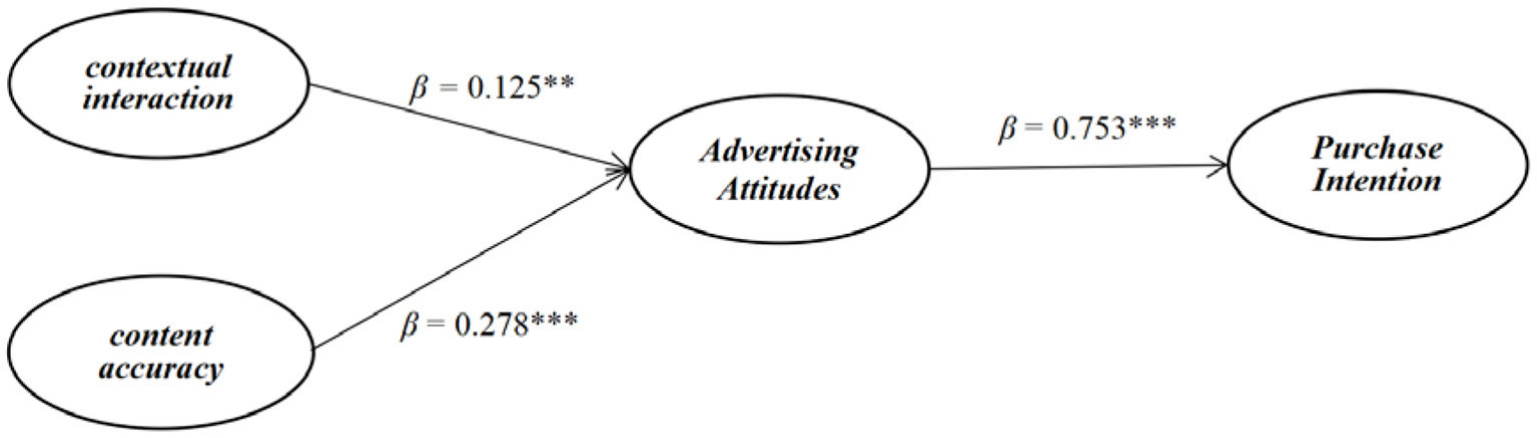
Structural analysis results. *Note:* Standardized coefficients. The numbers in parentheses are standard errors. **p < 0.01, ***p < 0.001.

The model fit indices revealed that the model had a good fit with the Comparative Fit Index (CFI) = 0.91, Incremental Fit Index (IFI) = 0.91, and Standard Root Mean Square Residual (SRMR) = 0.04, and Root Mean Square Error of Approximation (RMSEA) = 0.05. The results of the structural model are presented in Figure 2. The results showed that contextual interaction has a positive influence on advertising attitudes (β = 0.125, *t* = 3.286, *p* < 0.01), thus supporting H1. Content accuracy also positively influenced advertising attitudes (β = 0.278, *t* = 4.533, *p* < 0.001), supporting H2. Advertising attitudes were shown to have positive effects on purchase intention (β = 0.753, *t* = 31.952, *p* < 0.001), supporting H3. In conclusion, this study identified contextual interaction and content accuracy as important characteristics that motivate consumers to be attracted by contextual mobile advertising. In addition, these two characteristics of contextual mobile advertising are stimulated by positive advertising attitudes, which also effectively increase purchase intention.

### Moderation Analysis

To test moderation hypotheses, a two-way moderated regression was selected (Hair et al., 2006). Before running the moderation, the independent variables and moderating variables were mean-centered to avoid multi-collinearity (Aiken and West, 1991) and interaction terms were created for independent variables and moderating variables in the hypothesized relationships.

### Moderating Role of Extroversion

**Figure 3:**
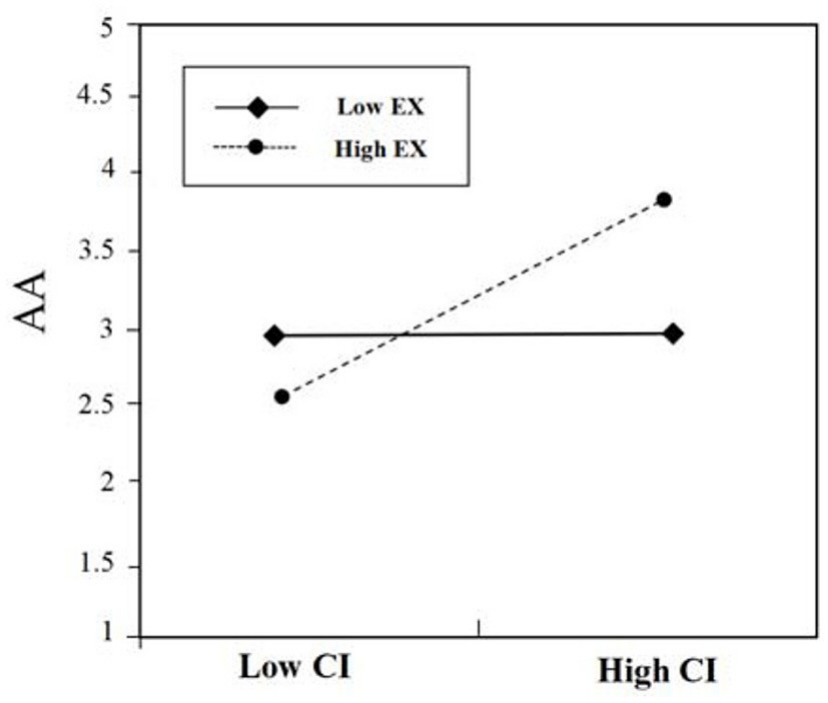
The moderating effect of EX on the relationship between CI and AA.

**Figure 4:**
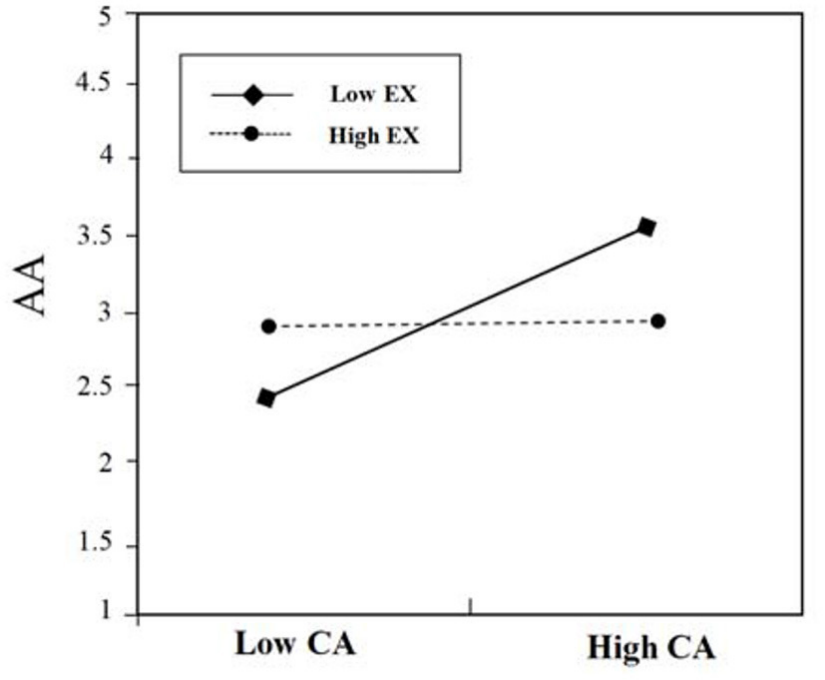
The moderating effect of EX on the relationship between CA and AA.

The analysis revealed (see Table 4) that control variables (e.g., gender, age, and education) did not explain any significant amount of variance in the advertising attitudes (Step 1). Step 2 shows that the direct effect of contextual interaction was significant on advertising attitudes (β = 0.674, *p* < 0.01), and the direct effect of content accuracy was significant on advertising attitudes (β = 0.727, *p* < 0.01). Step 3 shows the result of adding the interaction term (contextual interaction and extroversion; content accuracy and extroversion). As shown in Table 4 (step 3), the interactions (contextual interaction × extroversion; content accuracy × extroversion) were found significant for advertising attitudes (β = 0.137, *p* < 0.01; β = 0.203, *p* < 0.01). This analysis produced a significant main effect and a significant interaction effect (See Figures 3, 4 for details).

**Table 4:** Results for the main effect and moderated regression analyses.

**Predictors**	**Advertising attitudes**		**Advertising attitudes**	
	**β**	**Δ*R^**2**^***	**β**	**Δ*R^**2**^***
**Step1:**				
**Control variables**				
Gender	0.012		0.014	
Age	−0.047		−0.038	
Education	−0.089		−0.075	
**Step2:**				
**Main effects**				
Contextual interaction	0.674**			
Content accuracy			0.727**	
Extroversion	0.072*	0.376**	0.068*	0.445**
**Interaction terms**				
Contextual interaction × Extroversion	0.137**	0.014**		
Content accuracy × Extroversion			0.203**	0.017**

Note: *p < 0.05; **p < 0.01.

To further reveal the interaction effect, a simple slope test was performed. As shown in Figure 3, the results show when low extroversion (Low EX) (below one standard deviation), the influence of contextual interaction (CI) on advertising attitudes (AA) decreases, and the positive relationship between contextual interaction (CI) and advertising attitudes (AA) is not significant (β = 0.22, *ns*.); when high extroversion (High EX) (above one standard deviation), the positive relationship between contextual interaction (CI) and advertising attitudes (AA) is significant (β = 0.72, *p* < 0.001), that is, high extroversion is more sensitive to the changes of contextual interaction. Thus, those high in extroversion will show more positive advertising attitudes toward contextual mobile advertisements with high contextual interaction. Hypothesis 4a is supported.

As shown in Figure 4, the results show that when high extroversion (High EX) (above one standard deviation), the influence of content accuracy (CA) on advertising attitudes (AA) decreases, and the positive relationship between content accuracy (CA) and advertising attitudes (AA) is not significant (β = 0.25, *ns*.); when low extroversion (Low EX) (below one standard deviation), the positive relationship between content accuracy (CA) and advertising attitudes (AA) is significant (β = 0.68, *p* < 0.001), that is, low extroversion is more sensitive to the changes of content accuracy. Hypothesis 4b is not supported.

### Moderating Role of Neuroticism

**Figure 5:**
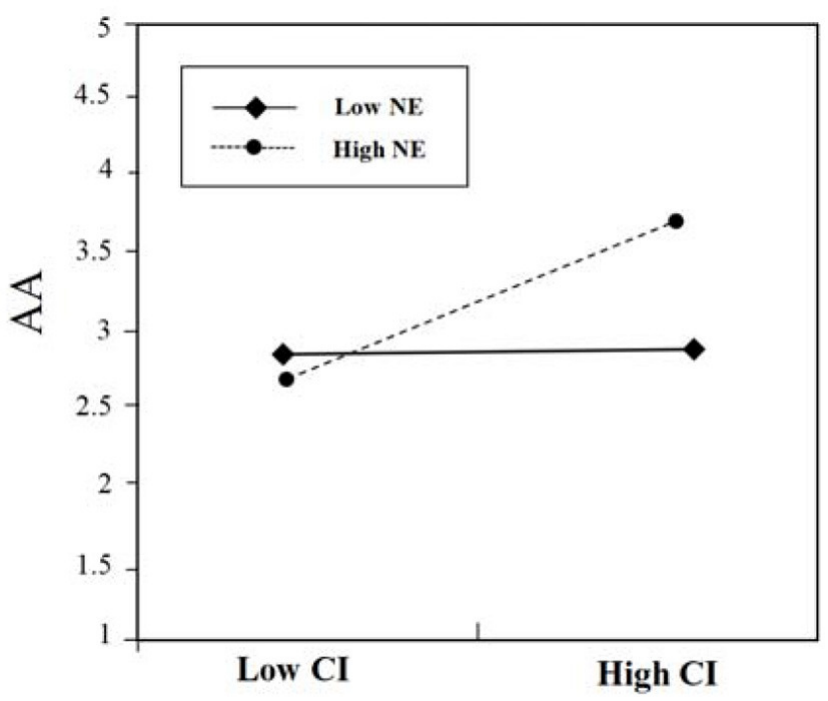
The moderating effect of NE on the relationship between CI and AA.

**Figure 6:**
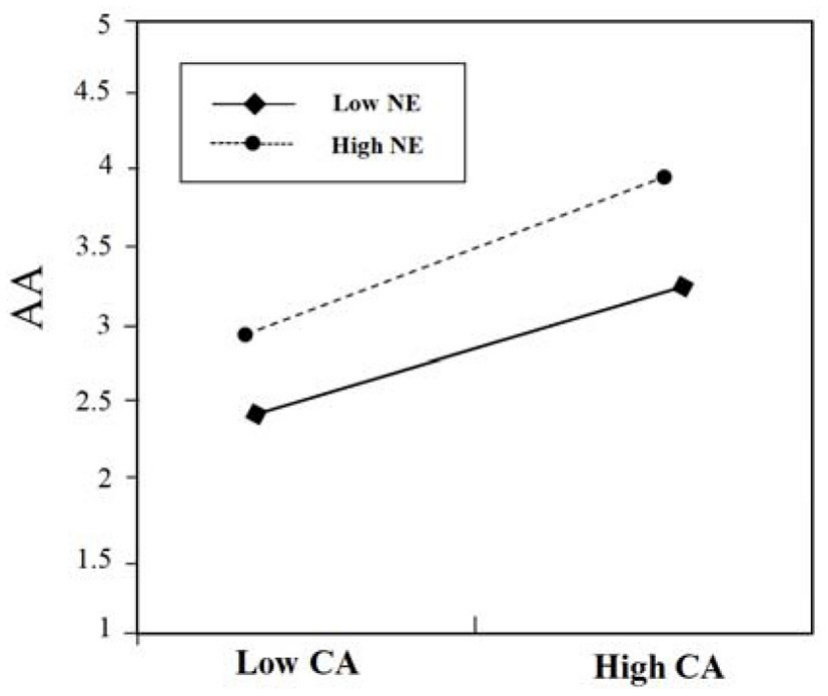
The moderating effect of NE on the relationship between CA and AA.

The analysis revealed (see Table 5) that control variables (e.g., gender, age, and education) did not explain any significant amount of variance in the advertising attitudes (Step 1). Step 2 shows that the direct effect of contextual interaction was significant on advertising attitudes (β = 0.563, *p* < 0.01), and the direct effect of content accuracy was significant on advertising attitudes (β = 0.697, *p* < 0.01). Step 3 shows the result of adding the interaction term (contextual interaction and neuroticism; content accuracy and neuroticism). As shown in Table 5 (step 3), the interactions (contextual interaction × neuroticism; content accuracy × neuroticism) were found significant for advertising attitudes (β = 0.125, *p* < 0.01; β = 0.221, *p* < 0.01). This analysis produced a significant main effect and a significant interaction effect (see Figures 5, 6 for details).

**Table 5:** Results for the main effect and moderated regression analyses.

**Predictors**	**Advertising attitudes**		**Advertising attitudes**	
	**β**	**Δ*R^**2**^***	**β**	**Δ*R^**2**^***
**Step1:**				
**Control variables**				
Gender	0.017		0.021	
Age	−0.038		−0.045	
Education	−0.075		−0.067	
**Step2:**				
**Main effects**				
Contextual interaction	0.563**			
Content accuracy			0.697**	
Neuroticism	0.069*	0.225**	0.072*	0.432**
**Interaction terms**				
Contextual interaction × Neuroticism	0.125**	0.018**		
Content accuracy × Neuroticism			0.221**	0.023**

Note: ^*^p < 0.05; ^**^p < 0.01.

To further reveal the interaction effect, a simple slope test was performed. As shown in Figure 5, the results show that in case of low neuroticism (Low NE) (below one standard deviation), the influence of contextual interaction (CI) on advertising attitudes (AA) decreases, and the positive relationship between contextual interaction (CI) and advertising attitudes (AA) is not significant (β = 0.24, *ns*.). When there is high neuroticism (High NE) (above one standard deviation), the positive relationship between contextual interaction (CI) and advertising attitudes (AA) is significant (β = 0.63, *p* < 0.001), that is, high neuroticism is more sensitive to the changes of contextual interaction. Thus, those high in neuroticism will show more positive advertising attitudes toward contextual mobile advertisements with high contextual interaction. Hypothesis 5a is supported.

As shown in Figure 6, the results show that when there is low neuroticism (Low NE) (below one standard deviation), the positive relationship between content accuracy (CA) and advertising attitudes (AA) is significant (β = 0.66, *p* < 0.001); when there is high neuroticism (High NE) (above one standard deviation), the positive relationship between content accuracy (CA) and advertising attitudes (AA) is also significant (β = 0.78, *p* < 0.001), that is, high neuroticism can promote the relationship of content accuracy and advertising attitudes. Low neuroticism has a weak moderating effect on the relationship between content accuracy and advertising attitudes. Thus, those high in neuroticism will show more positive advertising attitudes toward contextual mobile advertisements with high content accuracy. Hypothesis 5b is supported.

## Conclusion and Discussion

### Findings and Summary

The main purpose of this study was to investigate which characteristics of contextual mobile advertising evoke consumers' purchase intentions through advertising attitudes among community residents with the online shopping experience in China and to discuss how extroversion and neuroticism in personal traits moderate the relationship between significant characteristics of contextual mobile advertising and advertising attitudes. Through an extensive review of the prior literature and the status and issues in the development of contextual mobile advertising, this study concludes that contextual interaction and content accuracy can serve as stimuli that trigger positive consumer attitudes toward contextual mobile advertising and increase consumers' purchase intentions. Thus, contextual mobile advertising, featuring strong interactivity and high precision, is much easier to evoke consumer emotions and change consumer attitudes. This study further discussed the differences in perceptions and attitudes toward contextual mobile advertising characteristics between groups with extroversion traits and neuroticism traits. The findings showed that high extroversion and high neuroticism are more sensitive to the changes in contextual interaction, and low extroversion is more sensitive to the changes in content accuracy. High neuroticism can promote the relationship between content accuracy and advertising attitudes. Low neuroticism has a weak moderating effect on the relationship between content accuracy and advertising attitudes.

### Theoretical and Practical Implications

From the theoretical perspective, the influence of contextual mobile advertising characteristics on the purchase intention in this study is mainly based on two theories: First, the scene theory. Scene theory combines “scene” and “media,” and believes that the approach of new media will generate new scenes with fresh behaviors. With the booming of mobile Internet, the scene theory has been given new connotations, such as mobile devices, social media, big data, sensors, and location-based systems. These five technologies are closely related to scenario communication, and in turn, promote the communication effect. Based on the scene theory, this paper explored the mechanisms affecting consumers' advertising attitudes from the perspectives of contextual interaction and content accuracy, and then examined the influence of advertising attitudes on the purchase intention. Second, according to the Planned Behavior Theory, attitude is one of the essential factors influencing consumer behavior. Attitude is often considered an indicator in the study of mobile advertising effectiveness. Contextual mobile advertising characteristics not only reduce consumers' aversion and increase positive advertising attitudes but also induce consumers' purchase intentions and purchase behaviors. This study found that contextual mobile advertising characteristics play an important role in the practical application of stimulating advertising attitudes, and how to maximize consumer satisfaction through contextual stimulation would become a hot issue for future research in the domain of contextual mobile advertising. Meanwhile, this study explores how to maximize the communication value of advertising by enhancing contextual interaction and content accuracy. This study not only extends the research on mobile advertising but also further expands the antecedent variables of advertising attitudes. In addition, this study examines the moderating effects of extroversion traits and neuroticism traits on contextual mobile advertising characteristics and advertising attitudes from personality trait theory. From the practical perspective, the application of contextual mobile advertising is conditional and may affect individuals with different personality traits differently.

Overall, this study broadens the horizons of future research in exploring and studying the individual differences in contextual mobile advertising audiences. Contextual mobile advertising, rooted in location-based, big data, social media, and other technologies to achieve the accurate delivery of advertising, is changing the traditional bombing advertising patterns to reduce consumer advertising avoidance. Not only can contextual mobile advertising take consumers' current time, space, and psychological state into consideration entirely and cater to consumers' needs, but achieve higher advertising click rate, conversions, and higher ROI index of enterprises. Therefore, these findings can help advertisers improve the delivery efficiency of contextual mobile advertising and provide more personalized advertising recommendations to consumers (Xiang et al., 2021; Xu et al., 2021a,b; Wang et al., 2022). Moreover, even though contextual mobile advertising has many advantages, advertisers are still marketing their products or services which are hopefully the very content that consumers are focusing on to a wide variety of audiences through distribution networks. If advertisers can combine contextual mobile advertising characteristics (they are either highly interactive or highly accurate) and personality traits, to select matching scenarios for advertising placement, it will not only be consistent in scenarios and content, but truly personalized. As a result, contextual mobile advertising will no longer be a clutter of interfering information but directed to the “deep-rooted psychological sketching” and intrinsic demands of consumers.

### Limitations and Future Research

This study mainly discusses the internal mechanism of the influence of mobile advertising factors on the purchase intention, which enriches this research findings on mobile advertising, and lays a solid theoretical foundation for advertising practice, however, several limitations should be discussed. First, the methods are limited. This study collects and processes the data in the form of a questionnaire. Future research can use experimental methods or other research methods to demonstrate or supple the results of this study. Second, the research sample is limited. This study is deeply rooted in China's local background, and the sample data are mainly from Chinese consumers, which inevitably causes some regional limitations in the application of this study.

With the rapid development of mobile Internet, future research can be extended to the Asia-Pacific region or Euro-American region through international cooperation, to further pass the robustness test, re-estimating the results of this study by changing the “situation.” Finally, the measurement is limited. The scale of this study is summarized by referring to the previous research, without the development of the scale. Future research can modify and optimize the scale through the Grounded Theory and interview methods.

## Data Availability Statement

The original contributions presented in the study are included in the article/supplementary material, further inquiries can be directed to the corresponding author/s.

## Ethics Statement

The studies involving human participants were reviewed and approved by Ethics committee of Zhejiang gongshang University. The patients/participants provided their written informed consent to participate in this study.

## Author Contributions

YW and CX designed the study, conceived the manuscript, and drafted the manuscript. YW and SZ implemented the questionnaire survey. YW and ZZ were involved in revising the manuscript. All authors were involved in writing the manuscript and approve its final version.

## Funding

This research was supported by the project of China (Hangzhou) Cross-border E-commerce College (No. 2021KXYJ06), Philosophy and Social Science Foundation of Zhejiang Province (21NDJC083YB), National Natural Science Foundation of China (71702164), Natural Science Foundation of Zhejiang Province (LY20G010001), and the Contemporary Business & Trade Research Center of Zhejiang Gongshang University (No. XT202103, No. XT202105).

## Conflict of Interest

The authors declare that the research was conducted in the absence of any commercial or financial relationships that could be construed as a potential conflict of interest.

## Publisher's Note

All claims expressed in this article are solely those of the authors and do not necessarily represent those of their affiliated organizations, or those of the publisher, the editors and the reviewers. Any product that may be evaluated in this article, or claim that may be made by its manufacturer, is not guaranteed or endorsed by the publisher.

## Footnotes

^1^ https://report.iresearch.cn/report/202008/3632.shtml

^2^https://www.wjx.cn.
